# Robotic surgery for Crohn’s disease: multinational survey of colorectal surgeons

**DOI:** 10.1007/s11701-026-03336-2

**Published:** 2026-04-01

**Authors:** Ayesha Unadkat, Shobhit Arya, Aliki Rompou, George Giannos, Orsalia Mangana, Valerio Celentano

**Affiliations:** 1 Department of Surgery, Imperial College London, Exhibition Road, SW7 2AZ, London, England UK; 2 Department of Colorectal Surgery, Chelsea and Westminster Hospital, 369 Fulham Rd., SW10 9NH, London, England UK

**Keywords:** Robotic surgery, Inflammatory bowel disease, Crohn’s disease, International survey, Surgical standardisation

## Abstract

Robotic-assisted surgery is increasingly utilised in colorectal procedures, yet its adoption in Crohn’s disease (CD) remains limited. Challenges unique to CD—such as complex anatomy, prior operations, and inflammation—present substantial barriers to robotic integration, compounded by the absence of standardised operative strategies. We aimed to evaluate current global practices among colorectal surgeons regarding robotic ileocolic resection for CD, and to propose a standardised surgical planning framework to address variability in technique and perioperative planning. A structured multinational survey was iteratively developed using validated assessment tools, targeting colorectal consultants, fellows, and senior trainees and was disseminated via professional networks. Quantitative and qualitative responses regarding port placement, dissection strategies, intraoperative decision-making, and perceived barriers were analysed. Insights were synthesised with guidelines to inform the PRESERVED framework. The survey was disseminated via professional networks to colorectal surgeons in multiple countries. Survey responses (*n* = 30) from eight countries—predominantly UK-based (50%) and consultants (80%). Survey responses revealed low robotic uptake in IBD compared to colorectal cancer. Common challenges included adhesions, prior surgery, and multi-segment disease; robotic access was rarely a constraint (3.3%). Despite variable approaches, intracorporeal anastomosis and Pfannenstiel extraction were favoured. The PRESERVED framework—comprising nine modular perioperative phases—was developed to guide decision-making and mitigate CD-specific surgical risks. This study is the first multinational exploration of robotic surgical practices in CD. Despite limited uptake, expert insights underscore a growing interest in robotic approaches and the need for structured guidance. The PRESERVED framework offers an evidence-based, adaptable tool to support surgical planning and training. Further validation through consensus-building and multicentre prospective studies is warranted.

## Introduction

Crohn’s disease (CD) is a chronic inflammatory bowel disease (IBD), affecting millions of individuals globally and continues to present a significant health burden [1]. Despite advances in pharmacological treatments, including biologics and immunomodulatory agents, CD remains an incurable condition, with surgical care required when medical management fails. While surgery is often essential to prevent irreversible damage, it is technically demanding, particularly in the setting of chronic inflammation, mesenteric fibrosis, and prior surgical interventions.

The evolution of minimally invasive surgery (MIS) has significantly impacted CD surgical approaches. Laparoscopic techniques have been shown to result in reduced postoperative pain, faster recovery, and lower complication rates compared to open surgery [2]. These benefits are especially notable in CD, where reduced adhesion formation may lower the need for reoperations [3]. Despite these advantages, the application of MIS in IBD remains inconsistent.

Surgical management of CD presents unique difficulties due to involvement of multiple abdominal quadrants and intestinal segments, previous surgical interventions, malnutrition, immunosuppression, intra-abdominal sepsis, and complex fistulae. These factors can significantly increase the technical complexity and risk profile of operative procedures in CD.

Over the past two decades, evidence has accumulated supporting the use of robotic surgery in colorectal procedures, demonstrating outcomes comparable to or exceeding those of both laparoscopic and open techniques [4–6]. However, robotic surgery remains underutilised in IBD compared to its more widespread adoption in colorectal cancer surgery, where the use of robotic-assisted procedures has seen a marked increase [7].

Despite its potential, the uptake of robotic surgery in CD remains constrained by several barriers, including the lack of standardised protocols and unclear learning curve, which likely differs greatly from colorectal cancer surgery due to the anatomical challenges of IBD. This study aims to exploring the current practices of colorectal surgeons regarding robotic surgery in CD through a structured multinational survey. By doing so, we aim to lay the groundwork for a more standardised approach to surgical planning and propose a streamlined, evidence-based framework to support colorectal surgeons in managing this challenging patient population.

## Methods

Given the high prevalence of CD in the distal ileum, a structured, questionnaire-based survey was designed to focus specifically on robotic ileocolic resection—the most commonly performed procedure in this population [8].

Ethical approval was obtained from the Imperial College London Research Ethics Board, followed by subsequent departmental review at Chelsea and Westminster Hospital. Informed written consent was obtained from all participants prior to survey completion. All responses were collected anonymously.

The questionnaire was developed iteratively, drawing on thematic findings from our systematic review of robotic IBD surgery [9] to ensure conceptual alignment with the existing evidence base (see Appendix). Content validity was enhanced through expert review and refinement by experienced colorectal surgeons to ensure clinical relevance and completeness. Survey construction was informed by the Laparoscopic Competency Assessment Tool (LCAT) framework [10]. Design principles were further guided by established best practices in questionnaire development, including clarity of phrasing, response scaling, and construct validity, as described in the literature [11–13].

The survey was administered via Google Forms and disseminated to 60 eligible participants—colorectal consultants, fellows, and senior trainees with IBD experience on the international faculty, delegates list of our IBD centre webinar series and additional professional surgical networks in March–April 2025. Eligible participants included colorectal surgeons, fellows, and senior trainees with experience in robotic-assisted surgery for IBD. The survey included a combination of multiple-choice and open-ended items, structured to explore operative techniques, port placement strategies, decision-making frameworks, and perceived challenges in robotic surgery for CD. Domains were selected to address key gaps from the systematic review and refined with expert input.

The synthesis of phases and strategies within the PRESERVED framework was informed by triangulating insights from five sources: (i) findings from our systematic review on robotic IBD surgery (ii) analysis of survey responses, (iii) synthesis of European Crohn’s and Colitis Organisation (ECCO) guidelines [14], (iv) our institutional practice and experience, and (iv) expert video review of complex robotic CD dissections [15].

### Data analysis

All data were analysed using Microsoft Excel. Categorical variables were reported as frequencies with corresponding percentages. Qualitative responses were analysed descriptively.

## Results

### Respondent and institutional demographics

A total of 30 completed responses were collected. The majority of respondents were consultant or attending colorectal surgeons (80%, *n* = 24), with additional input from fellows (13.3%, *n* = 4) and senior surgical trainees or registrars (6.7%, *n* = 2). The respondent cohort was internationally diverse, representing eight countries. The highest proportion of responses originated from the United Kingdom (50%, *n* = 15), followed by Italy (*n* = 6), Brazil (*n* = 4), and Spain (*n* = 2), with single responses from the Philippines, Belgium, Greece, and Turkey.

**Fig. 1:**
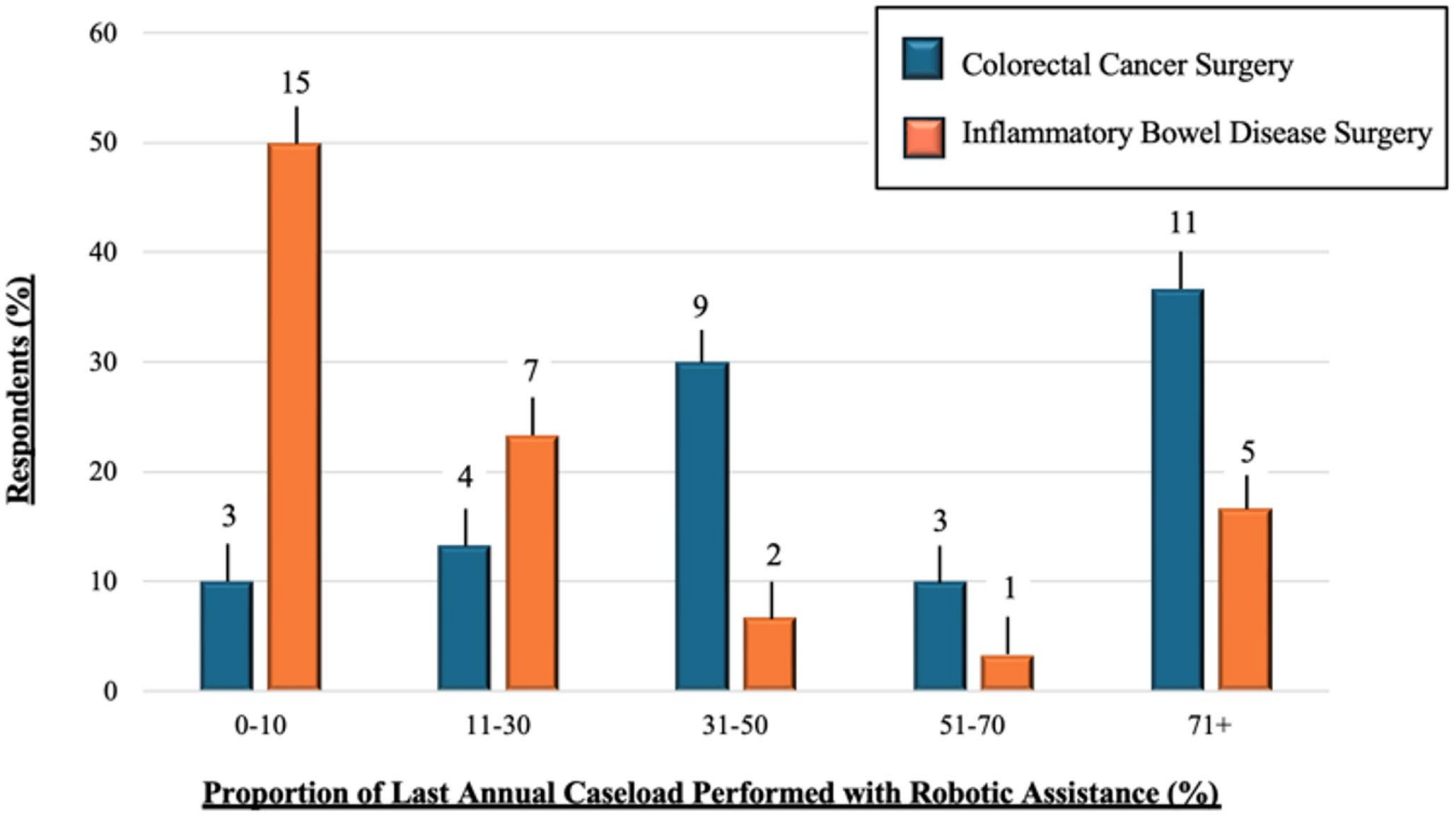
Distribution of robotic surgical case volumes in the last 12 months among survey respondents (*n* = 30). Respondents reported a greater frequency of robotic assistance in colorectal cancer surgery relative to inflammatory bowel disease surgery. Responses are grouped by indication: colorectal cancer surgery (blue) and inflammatory bowel disease surgery (orange). Absolute frequencies are indicated on each bar

Approximately 43.3% (*n* = 13) of participants reported affiliation with high-volume institutions, defined as centres performing ≥ 100 robotic colorectal resections annually. The da Vinci Xi platform was the most commonly utilised system (83.3%, *n* = 25). Despite access, only 16.7% (*n* = 5) of respondents indicated that robotic platforms were used in over 71% of IBD cases at their institution (Fig. 1). In contrast, 50% (*n* = 15) reported robotic assistance in fewer than 10% of IBD procedures. Robotic utilisation was notably higher in oncological contexts, with 36.7% (*n* = 11) of centres employing robotic techniques in over 71% of colorectal cancer surgeries.

## Operative strategy and port placement preferences

**Fig. 2:**
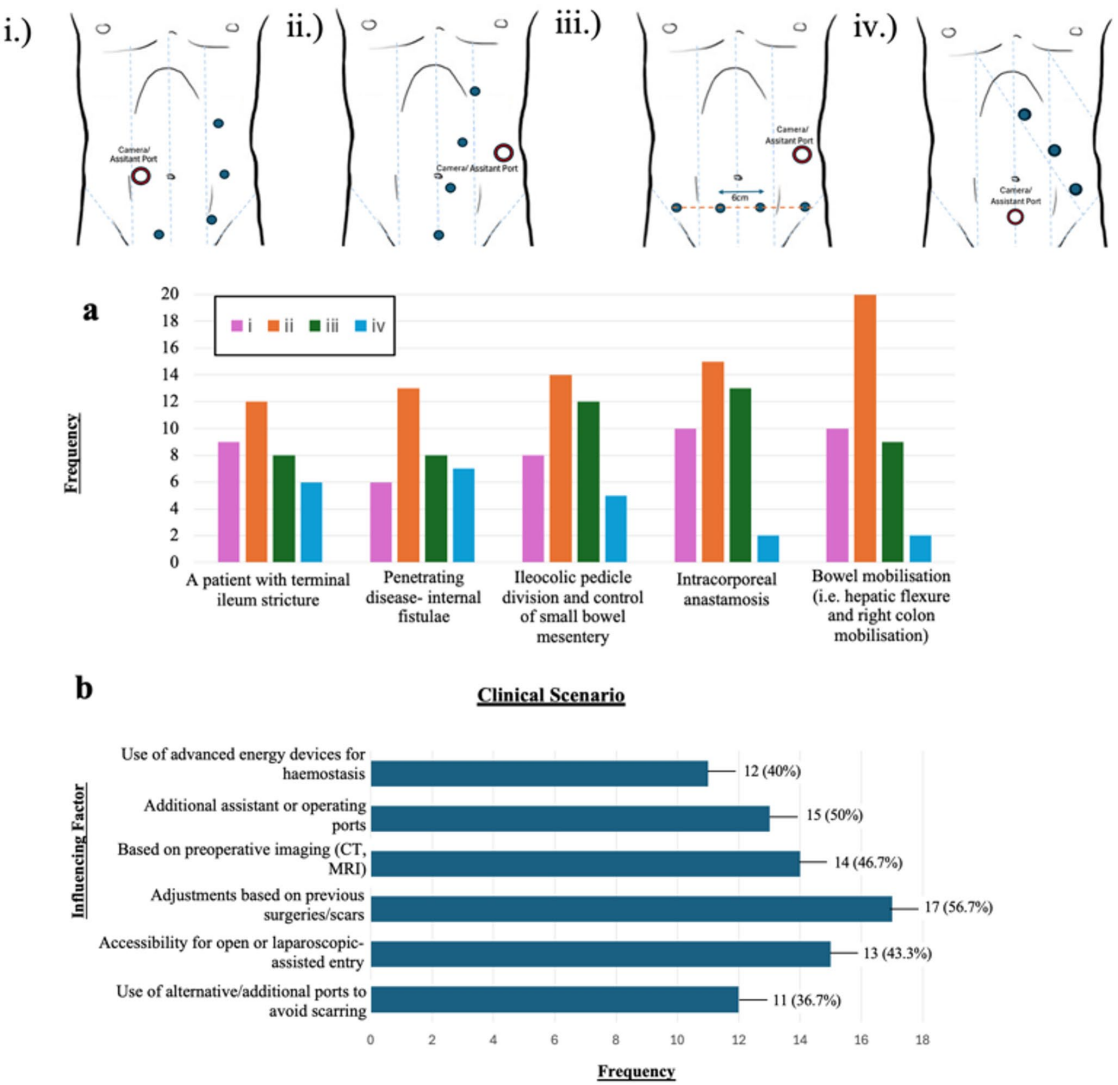
Preferred port placement configurations and influencing factors for robotic ileocaecal Crohn’s disease surgery, based on survey responses (*n* = 30). (**a**) Preferred port placement configurations across five defined clinical scenarios in Crohn’s disease surgery. Port placement strategy **ii** was most frequently favoured across all five scenarios. Four robotic port placement strategies (**i–iv**), representing the most commonly reported approaches identified through the systematic review and analysis of peer-reviewed robotic Crohn’s disease surgical videos (Giannos, Unadkat et al. 2025), were presented to participants, who selected their preferred configuration for each scenario. (**b**) Key factors influencing port placement decisions in patients with adhesions or mesenteric thickening reported by respondents. The most commonly cited consideration was the need for adjustments based on prior surgeries or scar distribution, followed by accessibility for open or laparoscopic-assisted entry. Multiple selections were permitted per respondent. Absolute frequencies are indicated on each bar, with corresponding percentages shown in brackets. *CT* indicates computed tomography; *MRI*, Magnetic resonance imaging

Trocar placement strategies varied significantly among respondents, one strategy—referred to here as Port Placement Strategy **ii**—emerged as the most frequently selected approach across a range of indications (Fig. 2a). Port configuration decisions were influenced by multiple intraoperative and patient-specific variables, with previous abdominal surgery and associated scarring cited as the most common determinant (56.7%, *n* = 17) (Fig. 2b).

Docking position was primarily guided by disease location, reported by 96.2% (*n* = 25) of respondents. Other considerations included patient positioning (40%, *n* = 12), assistant ergonomics (30%, *n* = 9), and proximity to key anatomic structures (30%, *n* = 9). Regarding specimen extraction, the Pfannenstiel incision was the preferred site (66.7%, *n* = 20), with the remainder opting for a periumbilical approach (33.3%, *n* = 10).

**Fig. 3:**
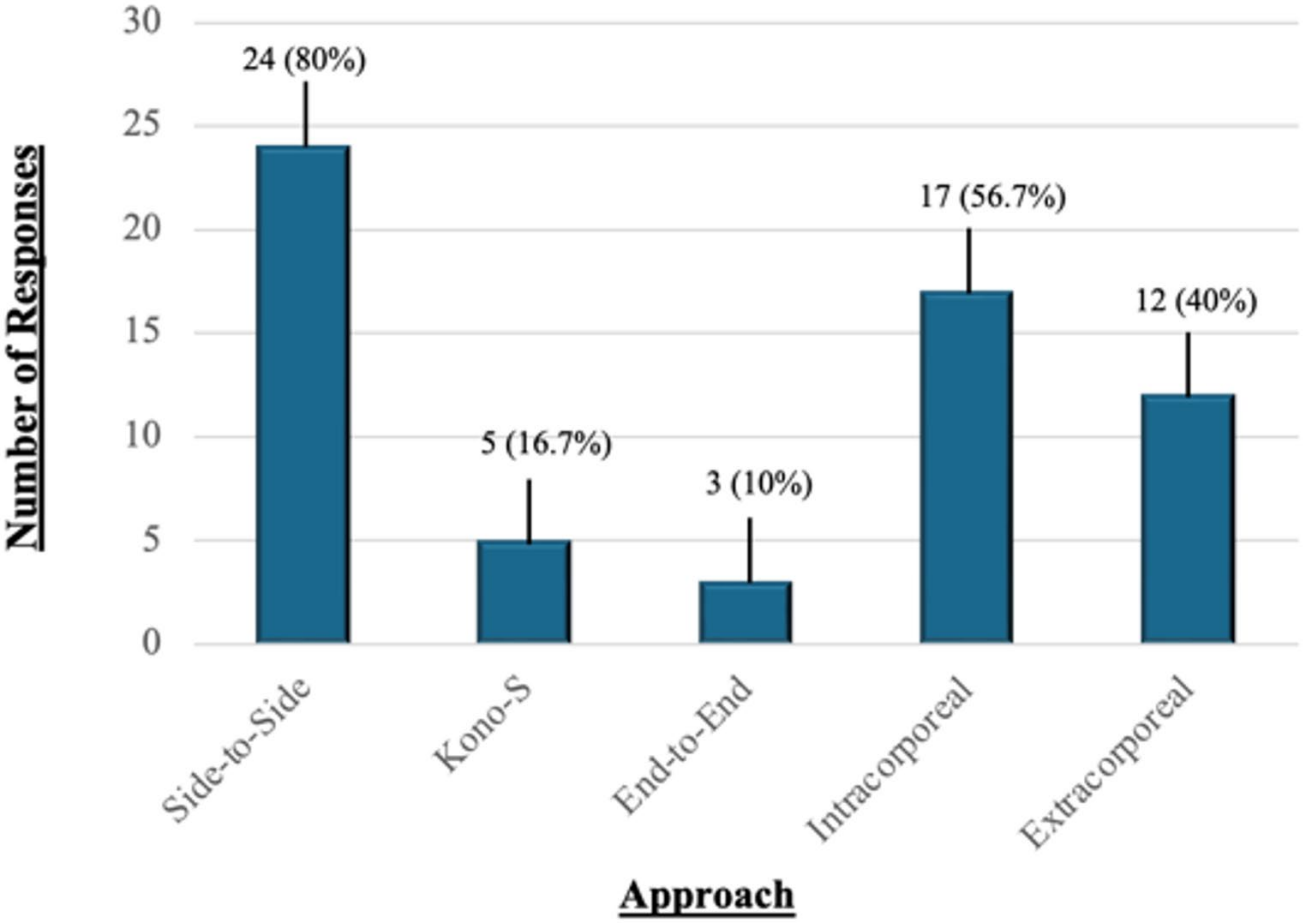
Distribution of preferred surgical techniques in robotic ileocaecal resection for Crohn’s disease among survey respondents (*n* = 30). Most frequently selected approaches included side-to-side anastomosis and intracorporeal resection. Multiple selections were permitted per respondent. Absolute frequencies are indicated on each bar, with corresponding percentages shown in brackets

In terms of dissection technique, half of the respondents (50%, *n* = 15) favoured a medial-to-lateral approach for bowel mobilisation and vascular control. A large majority (80%, *n* = 24) reported a preference for a side-to-side configuration for bowel anastomosis. Further details anastomotic strategies are illustrated in Fig. 3.

## Procedural challenges and contraindications

**Fig. 4:**
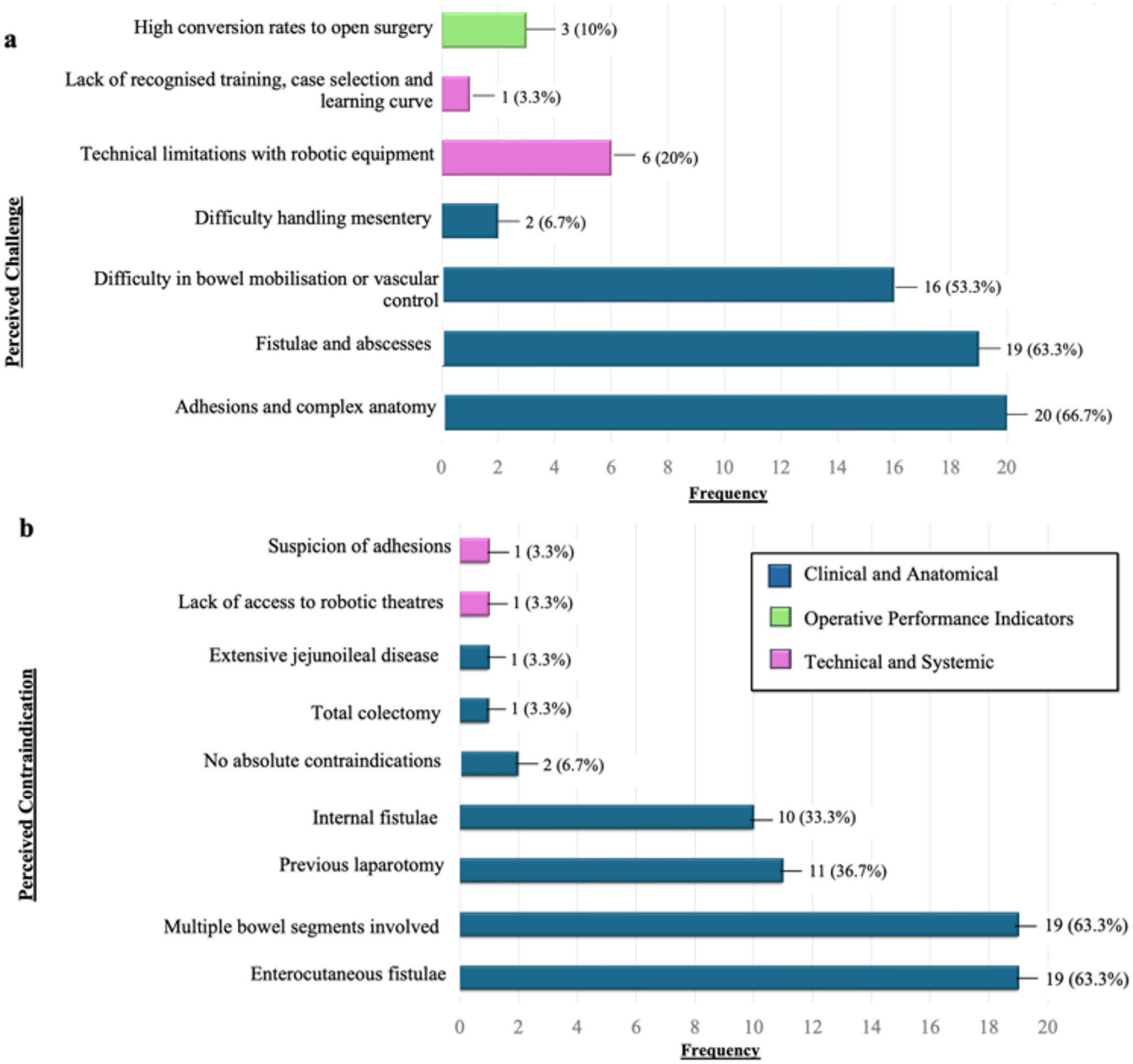
Perceived challenges and contraindications of robotic ileocaecal resection for Crohn’s disease among survey respondents (*n* = 30). The most commonly reported challenges and contraindications were of a clinical and anatomical nature. **(a)** Perceived challenges of robotic inflammatory bowel disease surgery. **(b)** Perceived contraindications for robotic inflammatory bowel disease surgery. Multiple selections were permitted per respondent. Responses are grouped into: Clinical and Anatomical Challenges (blue), Postoperative Outcomes (green), and Technical and Systemic Challenges (pink). Absolute frequencies are indicated on each bar, with corresponding percentages shown in brackets

The principal barriers to robotic surgery in CD were related to complex clinical and anatomical features. The most frequently cited intraoperative challenge was the presence of adhesions and distorted intra-abdominal anatomy (66.7%, *n* = 20), followed closely by inflammatory complications such as fistulae and abscesses (63.3%, *n* = 19) (Fig. 4a).

Free-text responses additionally highlighted system-level limitations, including restricted access to robotic platforms (*n* = 1) and the absence of formalised training pathways or structured case selection processes (*n* = 1). Commonly identified contraindications included the presence of enterocutaneous fistulae and extensive multi-segmental disease, both perceived as situations where robotic resection would be technically prohibitive (Fig. 4b).

We propose a novel way to approach this problem: the PRESERVED framework (presented in Table 1), synthesises this study’s insights with current guidelines to establish modular perioperative phases to act as a ‘checklist’ to navigate contraindications (e.g., reoperation, multi-segment disease) where robotic intervention may yield the greatest benefit. It outlines nine modular perioperative phases across preoperative, intraoperative and postoperative domains as informed by ECCO guidelines [14]. While some perioperative strategies apply broadly, the framework also considers CD-specific risks (e.g., malnutrition, catabolic states, and thromboembolism)—requiring multidisciplinary optimisation. The PRESERVED framework was developed to provide a structured and reproducible approach to robotic CD ileocolic resection outlining key operative steps designed to standardise surgical strategy and support training in complex inflammatory bowel disease surgery while accommodating the technical challenges in CD.

**Table 1:** Key components and rationale for the proposed ‘PRESERVED’ framework for robotic inflammatory bowel disease planning. Domains are grouped by preoperative, intraoperative and postoperative phases

		Domain	Description of Key Components	Rationale
Preoperative	*P*	Planning and Optimisation	Preoperative imaging and ileocolonoscopy, Integration of the Montreal Classification*, MDT discussion, disease mapping, nutritional optimisation, iron management, immunosuppression management, docking and port configuration planning	Enables personalised operative strategy; anticipates anatomical variation and complexity in reoperative or inflamed abdomens
	R	Reposition and Prophylaxis	Patient positioning (e.g., Trendelenburg, lithotomy), antibiotic and thromboembolism prophylaxis, pressure ulcer protection	Prevents positioning-related complications; optimises exposure given the fixed nature of robotic docking
Intraoperative	E	Establish Access	Pneumoperitoneum creation, port placement (robotic and assistant), arm spacing, quadrant planning.	Foundation of robotic workflow; ensures safe multi-quadrant reach, critical in subtotal colectomy or extensive Crohn’s disease
	S	Separate Adhesions & Identify Anatomy	Adhesiolysis, early recognition of ureters, retroperitoneum, vascular landmarks, and anatomical orientation in hostile or reoperative fields	Robotic precision facilitates safe navigation through dense adhesions and distorted planes common in IBD surgery
	E	Engage the Robot	Docking the robotic system, console transition, system calibration, team coordination	Smooth transition minimises delay and sets up for ergonomic, stable console work during critical dissection phases
	R	Release (Bowel Mobilisation)	Mobilisation of affected segments (medial or lateral approach), mesenteric handling, preservation of uninvolved bowel	Robotic articulation enhances precision in inflamed, fibrotic, or thickened mesentery, often seen in IBD
	V	Vascular Control	Identification and selective division of mesenteric vessels, haemostasis, avoidance of unnecessary devascularisation	Enables tailored, non-oncologic vascular strategies; robotic control aids in haemostasis of inflamed or oedematous tissues
	E	Excision and Anastomosis	Segmental resection, intracorporeal anastomosis, choice of extraction site (Pfannenstiel vs. midline), closure technique	Robotic suturing supports precise anastomosis; intracorporeal techniques may enhance cosmesis and reduce wound complications
Postoperative	D	Debrief (Outcomes and Learning)	Team-based review of intraoperative challenges, deviations from plan, outcomes, and takeaways. Reporting complications by Clavien-Dindo Classification†. Adherence to ERAS pathway including optimised nutrition, analgesia, mobilisation and extended thromboprophylaxis	Reinforces reflective practice, supports training, and provides data for quality improvement and standardised audit across centres

*MDT *indicates multi-disciplinary team;* IBD, *inflammatory bowel disease;* RAS, *enhanced recovery after surgery

*Disease phenotype defined stratified to the Montreal classification system

^†^Postoperative complications stratified according to the Clavien-Dindo Classification

## Discussion

This survey provides a new insight into robotic ileocolic resection practices among colorectal surgeons, offering a meaningful counterpoint to the largely US-centric literature [16, 17]. An important consideration of this study given its largely UK-based cohort is the pronounced geographical disparity in robotic platform access—an issue underrepresented in prior analyses. In lower-resource healthcare settings, the considerable capital investment required for robotic infrastructure often results in restricted availability, delayed access, or complete inaccessibility, frequently reflecting underlying socioeconomic inequities. Consequently, colorectal surgery remains centralised to high-volume, tertiary referral centres, limiting generalisability and perpetuating a literature bias towards well-resourced institutions [18]. Notably, despite only 3.3% of respondents citing lack of platform access as a major challenge (Fig. 4b), half reported performing robotic surgery in ≤ 10% of their IBD cases (Fig. 1). This suggests that while access to robotic systems may exist, consistent integration into routine IBD care remains limited—possibly due to institutional prioritisation of cancer pathways, inadequate case volumes, or lack of structured training.

Our findings also underscore the slower adoption of robotic techniques in IBD surgery compared with oncological colorectal surgery. In this cohort, robotic utilisation in IBD surgery was markedly lower than in colorectal cancer, a discrepancy likely driven by the distinct technical demands of CD. These include dense intra-abdominal adhesions, distorted anatomy from prior operations, inflamed or friable mesentery, and the need for more extensive dissection. As such, direct transference of oncological robotic techniques to IBD surgery may not be feasible. This may explain why key procedural steps such as vascular control and fistula management—typically less problematic in cancer cases—emerge as operative challenges in our cohort. Nevertheless, several robotic advantages demonstrated in colorectal cancer surgery—such as enhanced pelvic visualisation, improved nerve preservation, and refined intracorporeal stapling—may offer particular value in younger IBD populations, where considerations of fertility, cosmesis, and long-term function are especially pertinent [19, 20]. The majority of respondents (80%) reported a preference for side-to-side intracorporeal anastomosis (ICA) (Fig. 3), consistent with a growing body of literature supporting ICA’s benefits, including reduced bowel traction, improved visualisation, potential for off-midline extraction, and expedited postoperative recovery [21–23]. Furthermore, this approach aligns with ECCO recommendations, whereas the Kono-S technique, though also guideline-recommended and supported by studies suggesting lower rates of endoscopic recurrence, remained with low uptake in our cohort (16.7%) [14]. Its limited adoption may be attributable to technical complexity, the steep learning curve, and institutional inertia. The predominance of extracorporeal anastomosis in some centres further reflects the conservative integration of newer techniques within robotic platforms and highlights a broader trend towards risk-averse adoption during early implementation. Port placement strategies varied widely, highlighting the absence of a universally accepted configuration (Fig. 2). Nonetheless, Port Strategy ii emerged as the most commonly favoured, largely due to its compatibility with suprapubic extraction and use of a Pfannenstiel incision. This configuration aligns with existing data demonstrating reduced hernia incidence, improved aesthetic outcomes, and lower intraoperative blood loss associated with suprapubic access [24–26].

The medial-to-lateral dissection approach involves early entry into the mesenteric plane at the root of the ileocolic vessels, allowing vascular control and mobilisation to proceed along embryological tissue planes before continuing laterally towards the abdominal wall. This strategy may facilitate safer dissection in robotic surgery by enabling early identification of key vascular structures and controlled mesenteric division. Although a medial-to-lateral dissection approach predominated in ileocolic resections, emerging evidence suggests potential benefits of a lateral-to-medial technique in patients with severe inflammation, as it may limit mesenteric traction and reduce manipulation of retroperitoneal structures [27]. These divergent strategies emphasise the need for a nuanced, patient-specific approach informed by disease phenotype, operative history, and anatomical variation. An alternative strategy described by respondents was the bottom-up (caudal-to-cranial) approach, in which dissection begins at the terminal ileum or caecum and proceeds proximally along the mesentery. This approach may be employed in selected cases where dense inflammatory adhesions or distorted anatomy make medial access to the mesenteric root technically challenging.

Interestingly, conversion to open surgery—often highlighted as a key performance metric in robotic series [27]—was reported as a major challenge by only a minority (10%) of respondents (Fig. 4a). This may reflect growing operator familiarity with robotic platforms and a shift toward evaluating surgical success based on broader metrics, such as complication rates, operative efficiency, and functional recovery. However, this discrepancy between published metrics and clinical sentiment remains poorly understood and merits further investigation.

In line with existing evidence, survey respondents identified multi-segment disease, previous laparotomy, and intra-abdominal sepsis (i.e., fistulae or abscesses) as relative contraindications to robotic surgery (Fig. 4b). These findings are consistent with published data indicating substantially higher conversion rates—up to 37%—in complex CD cases, compared to approximately 14% in less involved presentations [28]. However, this is increasingly challenged by emerging series demonstrating the feasibility and safety of robotic reoperations in IBD. Several studies report stable conversion rates, perioperative morbidity, and mortality when employing tailored strategies—such as preoperative imaging-informed port mapping, safe access planning, and dual docking techniques for multiquadrant access [29, 30]. Our respondents similarly cited prior surgical history, safe access, and imaging as key determinants of operative planning, reinforcing the growing consensus around patient-specific surgical design. These findings affirm that prior operations and complicated disease phenotypes do not constitute absolute contraindications to robotic surgery when approached through meticulous perioperative planning and standardised recovery protocols [31–33]. By integrating imaging-informed access, positioning, and disease mapping with CD-specific risk mitigation, PRESERVED aims to support supports the safe expansion of robotic surgery into these more challenging disease phenotypes.

Finally, there are limitations to consider when interpreting the results of our survey. While most survey participants were senior consultants from tertiary referral centres—raising the possibility of selection bias—their insights remain highly relevant. Due to its complexity, robotic IBD surgery is largely confined to such specialised environments, and these surgeons are uniquely positioned to inform curriculum development and protocol refinement. Additionally, the inclusion of multiple responses from single institutions could overrepresent certain practices or perspectives, warranting cautious interpretation of generalisability.

Additionally, a limitation of this study relates to the geographical distribution of respondents. Although the survey included colorectal surgeons from multiple countries, approximately half of participants were based in the United Kingdom. This may reflect differences in regional training pathways and service organisation. Within the UK, robotic colorectal surgery is often delivered through designated institutional robotic centres, with access to robotic platforms and training opportunities concentrated within specific units rather than uniformly distributed across all hospitals. Training is therefore typically undertaken through structured institutional programmes or fellowship-level exposure, which may influence both surgeon experience and patterns of case selection. Consequently, the perspectives captured in this survey may be more reflective of practice within healthcare systems with established robotic training infrastructure. Future studies incorporating broader international representation would help to further validate the findings and improve generalisability across different healthcare settings.

Nonetheless, this survey represents the first multinational exploration of robotic IBD surgery and provides valuable preliminary insight into evolving practice patterns. High-volume centres, in particular, are well positioned to lead future collaborative efforts in research, training, and protocol standardisation.

## Future directions

Potential for the PRESERVED framework extends beyond care principles by reflecting surgical education models, which have extensive evidence of accelerating learning, decision-making, and intraoperative communication [34, 35]. It aims to enhance reproducibility in reporting, auditing, and planning while preserving case-specific flexibility. Although currently conceptual, its refinement requires external validation through Delphi consensus supported by qualitative interviews involving multidisciplinary stakeholders.

Training remains a key near-future focus as robotic platform use expands, particularly given the underexplored learning curve. High-fidelity intraoperative recording and telemonitoring offer valuable tools to accelerate and standardise skill acquisition. However, reliance on unregulated, variable-quality educational videos in IBD impedes consistent competency development [36]. Extending the PRESERVED framework to formalise presentation of educational materials, alongside validated assessment tools like the GEARS scale and LAP-VEGaS checklist, could improve training resource quality [37].

As a pathway to longer-term clinical impact, stepwise validation should begin with single-centre audits, expanding to multicentre prospective pilot studies across diverse geographic and socioeconomic settings to address this literature gap whilst assessing clinician acceptability, adherence, and utility. Proof-of-concept studies must precede evidence-based, procedure-specific adaptations.

## Conclusion

This study provides a perspective on the evolving practices of robotic-assisted surgery in CD, highlighting variation in operative strategies and barriers largely related to anatomical complexity. We propose the PRESERVED framework as a novel, evidence-based framework to guide surgical planning, and standardise operative reporting in robotic CD surgery. Future efforts should focus on validating this model through Delphi consensus and prospective trials, with the aim of improving patient outcomes and facilitating greater reproducibility in robotic CD surgery.

## Data Availability

No datasets were generated or analysed during the current study.

## References

[1] Alatab S, Sepanlou SG, Ikuta K et al (2020) GBD 2017 Inflammatory Bowel Disease Collaborators. The global, regional, and national burden of inflammatory bowel disease in 195 countries and territories, 1990–2017: a systematic analysis for the Global Burden of Disease Study 2017. Lancet Gastroenterol Hepatol 5(1):17–30 PMID:31648971. 10.1016/S2468-1253(19)30333-4 31648971 10.1016/S2468-1253(19)30333-4 PMC7026709

[2] Pak SJ, Kim YI, Yoon YS, Lee JL, Lee JB, Yu CS (2021) Short-term and long-term outcomes of laparoscopic vs open ileocolic resection in patients with Crohn’s disease: propensity-score matching analysis. World J Gastroenterol 27(41):7159–7172. 10.3748/wjg.v27.i41.7159PMID:34887635; PMCID:PMC8613650 34887635 10.3748/wjg.v27.i41.7159 PMC8613650

[3] Duricova D (2017) What can we learn from epidemiological studies in inflammatory bowel disease? Dig Dis 35(1–2):69–73. 10.1159/000449086 28147360 10.1159/000449086

[4] Zaman A, Cavallaro P, Wexner SD (2020) Robotic surgery in colorectal practice: current perspectives. Clin Colon Rectal Surg 33(5):317–324. 10.1055/s-0040-1701475

[5] Baik SH, Kwon HY, Kim JS, Hur H, Min BS, Kim NK (2019) Robotic surgery for rectal cancer: an overview. Surg Endosc 33(6):1649–1655. 10.1007/s00464-019-06741-5

[6] Moghadamyeghaneh Z, Phelan M, Smith BR, Stamos MJ (2015) Outcomes of open, laparoscopic, and robotic abdominoperineal resections in patients with rectal cancer. Dis Colon Rectum 58(12):1123–1129. 10.1097/DCR.0000000000000475 26544808 10.1097/DCR.0000000000000475

[7] Taylor JC, Burke D, Iversen LH, YCR BCIP Study Group et al (2024) Minimally invasive surgery for colorectal cancer: benchmarking uptake for a regional improvement programme. Clin Colorectal Cancer 23(4):382–91e1. 10.1016/j.clcc.2024.05.013 39004595 10.1016/j.clcc.2024.05.013

[8] Anto VP, Dawes AJ, Vrees M, Watson AR, Lightner AL. Surgical management of inflammatory bowel disease. R I, Med J (2013) 2022;105(10):25–30. PMID:36413448; PMCID:PMC11554358 PMC11554358 36413448

[9] Unadkat A, Arya S, Rompou A, Celentano V. Standardisation in robotic surgery for inflammatory bowel disease: a systematic review. J Robot Surg. 2026;20(1):274. Published 2026 Feb 21. doi:10.1007/s11701-026-03194-y 10.1007/s11701-026-03194-y PMC12923402 41721185

[10] Mackenzie H, Ni M, Miskovic D, Motson RW, Gudgeon M, Khan Z et al (2015) Clinical validity of consultant technical skills assessment in the English National Training Programme for Laparoscopic Colorectal Surgery. Br J Surg 102(8):991–997. 10.1002/bjs.9828 25994456 10.1002/bjs.9828

[11] Gehlbach H, Artino AR Jr (2018) The survey checklist (manifesto). Acad Med 93(3):360–366. 10.1097/ACM.0000000000001839 29210756 10.1097/ACM.0000000000002083

[12] Krosnick JA, Presser S (2010) Question and questionnaire design. In: Marsden PV, Wright JD (eds) Handbook of Survey Research, 2nd edn. Emerald Group Publishing, Bingley (UK), pp 263–314

[13] Brasel K, Haider A, Haukoos J (2020) Practical guide to survey research. JAMA Surg 155(4):351–352. 10.1001/jamasurg.2019.4401 31995149 10.1001/jamasurg.2019.4401

[14] Adamina M, Bonovas S, Raine T et al (2020) ECCO Guidelines on therapeutics in Crohn’s disease: surgical treatment. J Crohns Colitis 14(2):155–168. 10.1093/ecco-jcc/jjz187 31742338 10.1093/ecco-jcc/jjz187

[15] Giannos G, Unadkat A et al (2025) Robotic surgery for Crohn’s disease: educational value and quality of online video resources. Surg Endosc. Manuscript submitted for publication 10.1007/s00464-025-12385-x 41261249

[16] Zaman S, Mohamedahmed AY, Abdelrahman W, Abdalla HE, Wuheb AA, Issa MT, Faiz N, Yassin NA (2024) Minimally invasive surgery for inflammatory bowel disease: a systematic review and meta-analysis of robotic versus laparoscopic surgical techniques. J Crohns Colitis 18(8):1342–1355. 10.1093/ecco-jcc/jjae037 38466108 10.1093/ecco-jcc/jjae037 PMC11324345

[17] Barash Y, Klang E, Tau N, Ben-Horin S, Mahajna H, Levartovsky A, Arebi N, Soffer S, Kopylov U (2021) Evolution of inflammatory bowel disease research from a bird’s-eye perspective: a text-mining analysis of publication trends and topics. Inflamm Bowel Dis 27(3):434–439. 10.1093/ibd/izaa091 32440691 10.1093/ibd/izaa091

[18] Ghandour O, Karimi A, Murphy J. Could colorectal cancer surgery in England benefit from centralisation? A retrospective nationwide correlative study. Br J Surg. 2023;110(Suppl 7):vii9. doi:10.1093/bjs/znad258.033.

[19] Bartels SAL, D’Hoore A, Cuesta MA, Bensdorp AJ, Lucas C, Bemelman WA (2012) Significantly increased pregnancy rates after laparoscopic restorative proctocolectomy: a cross-sectional study. Ann Surg 256(6):1045–1048. 10.1097/SLA.0b013e3182704be9 22609840 10.1097/SLA.0b013e318250caa9

[20] Kim JY, Kim NK, Lee KY, Hur H, Min BS, Kim JH (2012) A comparative study of voiding and sexual function after total mesorectal excision with autonomic nerve preservation for rectal cancer: laparoscopic versus robotic surgery. Ann Surg Oncol 19(8):2485–2493. 10.1245/s10434-012-2262-1 22434245 10.1245/s10434-012-2262-1

[21] Renshaw S, Silva IL, Hotouras A et al (2018) Perioperative outcomes and adverse events of robotic colorectal resections for inflammatory bowel disease: a systematic literature review. Tech Coloproctol 22(3):161–177. 10.1007/s10151-018-1762-9 29546470 10.1007/s10151-018-1766-5 PMC5862938

[22] Genova P, Pantuso G, Cipolla C, Latteri MA, Abdalla S, Paquet JC (2020) Laparoscopic versus robotic right colectomy with extracorporeal or intracorporeal anastomosis: a systematic review and meta-analysis. Langenbecks Arch Surg. 10.1007/s00423-020-01985-x 32902707 10.1007/s00423-020-01985-x

[23] Calini G, Abdalla S, Abd El Aziz MA et al (2022) Intracorporeal versus extracorporeal anastomosis for robotic ileocolic resection in Crohn’s disease. J Robot Surg 16(3):601–609. 10.1007/s11701-021-01283-8 34313950 10.1007/s11701-021-01283-8

[24] Samia H, Lawrence J, Nobel T, Stein S, Champagne BJ, Delaney CP (2013) Extraction site location and incisional hernias after laparoscopic colorectal surgery: should we be avoiding the midline? Am J Surg 205(3):264–267. 10.1016/j.amjsurg.2012.02.022 23375702 10.1016/j.amjsurg.2013.01.006

[25] Orcutt ST, Balentine CJ, Marshall CL, Robinson CN, Anaya DA, Artinyan A et al (2012) Use of a Pfannenstiel incision in minimally invasive colorectal cancer surgery is associated with a lower risk of wound complications. Tech Coloproctol 16(2):127–132. 10.1007/s10151-011-0810-8 22350173 10.1007/s10151-012-0808-7

[26] Gunnells D, Cannon J (2021) Robotic surgery in Crohn’s disease. Clin Colon Rectal Surg 34(5):286–291. 10.1055/s-0041-1729862 34512197 10.1055/s-0041-1729862 PMC8426043

[27] Miller AT, Berian JR, Rubin M et al (2012) Robotic-assisted proctectomy for inflammatory bowel disease: a case-matched comparison of laparoscopic and robotic technique. J Gastrointest Surg 16(4):587–594. 10.1007/s11605-011-1701-3 21964583 10.1007/s11605-011-1692-6

[28] Goyer P, Alves A, Bretagnol F, Bouhnik Y, Valleur P, Panis Y (2009) Impact of complex Crohn’s disease on the outcome of laparoscopic ileocecal resection: a comparative clinical study in 124 patients. Dis Colon Rectum 52(2):205–210. 10.1007/DCR.0b013e31819c9c08 19279413 10.1007/DCR.0b013e31819c9c08

[29] Abd El Aziz MA, Abdalla S, Calini G, Saeed H, D’Angelo A-L, Behm KT et al (2023) Robotic redo ileocolonic resection for Crohn’s disease: a preliminary report from a tertiary care center. Dis Colon Rectum 66(8):1095–1101. 10.1097/DCR.0000000000002380 36538722 10.1097/DCR.0000000000002380

[30] Ferrari L, Nicolaou S, Adams K (2024) Implementation of a robotic surgical practice in inflammatory bowel disease. J Robot Surg 18(1):57. 10.1007/s11701-023-01750-4 38281204 10.1007/s11701-023-01750-4

[31] Lovely JK, Maxson PM, Jacob AK et al (2012) Case-matched series of enhanced versus standard recovery pathway in minimally invasive colorectal surgery. Br J Surg 99(2):120–126. 10.1002/bjs.7692 21948187 10.1002/bjs.7692

[32] Spanjersberg WR, Reurings J, Keus F, van Laarhoven CJ (2011) Fast track surgery versus conventional recovery strategies for colorectal surgery. Cochrane Database Syst Rev 2CD007635. 10.1002/14651858.CD007635.pub2 10.1002/14651858.CD007635.pub2 PMC13061361 21328298

[33] Varadhan KK, Neal KR, Dejong CH, Fearon KC, Ljungqvist O, Lobo DN (2010) The enhanced recovery after surgery (ERAS) pathway for patients undergoing major elective open colorectal surgery: a meta-analysis of randomized controlled trials. Clin Nutr 29(3):434–440. 10.1016/j.clnu.2010.01.004 20116145 10.1016/j.clnu.2010.01.004

[34] Anderson CI, Gupta RN, Larson JR et al (2013) Impact of objectively assessing surgeons’ teaching on effective perioperative instructional behaviors. JAMA Surg 148(10):915–922. 10.1001/jamasurg.2013.2144 23945792 10.1001/jamasurg.2013.2144

[35] Catchpole K, Perkins C, Bresee C et al (2016) Safety, efficiency and learning curves in robotic surgery: a human factors analysis. Surg Endosc 30(9):3749–3762. 10.1007/s00464-015-4671-2 26675938 10.1007/s00464-015-4671-2 PMC13463350

[36] Celentano V, Browning M, Hitchins C, Giglio MC, Coleman MG (2017) Training value of laparoscopic colorectal videos on the World Wide Web: a pilot study. Surg Endosc 31(11):4496–4504. 10.1007/s00464-017-5504-2 28378076 10.1007/s00464-017-5504-2

[37] Goh AC, Goldfarb DW, Sander JC, Miles BJ, Dunkin BJ (2012) Global evaluative assessment of robotic skills: validation of a clinical assessment tool to measure robotic surgical skills. J Urol 187(1):247–252. 10.1016/j.juro.2011.09.032 22099993 10.1016/j.juro.2011.09.032

